# Day 100 Peripheral Blood Absolute Lymphocyte/Monocyte Ratio and Survival in Classical Hodgkin's Lymphoma Postautologous Peripheral Blood Hematopoietic Stem Cell Transplantation

**DOI:** 10.1155/2013/658371

**Published:** 2013-04-28

**Authors:** Luis F. Porrata, David J. Inwards, Stephen M. Ansell, Ivana N. Micallef, Patrick B. Johnston, William J. Hogan, Svetomir N. Markovic

**Affiliations:** Division of Hematology, Department of Medicine, Mayo Clinic, 200 First Street SW, Rochester, MN 55905, USA

## Abstract

Day 100 prognostic factors of postautologous peripheral blood hematopoietic stem cell transplantation (APBHSCT) to predict clinical outcome in classical Hodgkin lymphoma (cHL) patients have not been evaluated. Thus, we studied if the day 100 peripheral blood absolute lymphocyte/monocyte ratio (Day 100 ALC/AMC) affects clinical outcomes by landmark analysis from day 100 post-APBHSCT. Only cHL patients achieving a complete remission at day 100 post-APBHSCT were studied. From 2000 to 2010, 131 cHL consecutive patients qualified for the study. The median followup from day 100 was 4.1 years (range: 0.2–12.3 years). Patients with a Day 100 ALC/AMC ≥ 1.3 experienced superior overall survival (OS) and progression-free survival (PFS) compared with Day 100 ALC/AMC < 1.3 (from day 100: OS, median not reached versus 2.8 years; 5 years OS rates of 93% (95% CI, 83%–97%) versus 35% (95% CI, 19%–51%), resp., *P* < 0.0001; from day 100: PFS, median not reached versus 1.2 years; 5 years PFS rates of 79% (95% CI, 69%–86%) versus 27% (95% CI, 14%–45%), resp., *P* < 0.0001). Day ALC/AMC ratio was an independent predictor for OS and PFS. Thus, Day 100 ALC/AMC ratio is a simple biomarker that can help to assess clinical outcomes from day 100 post-APBHSCT in cHL patients.

## 1. Introduction

Day 100 after stem cell transplantation is currently the standard of care first follow-up visit to assess response after stem cell transplantation. In allogeneic stem cell transplantation, several day 100 prognostic factors have been studied to predict clinical outcomes including day 100 absolute lymphocyte count (ALC) [1, 2], day 100 absolute monocyte count (AMC) [1, 2], day 100 platelet count [3], graft-versus-host disease [4], and day 100 full donor chimerism [5]. In autologous stem cell transplantation, multiple myeloma documented minimal residual disease at day 100 was associated with inferior prognosis [6, 7] However, prognostic factors to assess prognosis for classical Hodgkin's lymphoma (cHL) achieving a complete remission at day 100 postautologous peripheral blood hematopoietic stem cell transplantation (APBHSCT) have not been evaluated. We previously reported that the peripheral blood absolute lymphocyte/monocyte count ratio at diagnosis (ALC/AMC-DX), as a surrogate biomarker of host immunity (i.e., ALC) and tumor microenvironment (i.e., AMC), was a prognostic factor for overall survival (OS), lymphoma-specific survival (LSS), progression-free survival (PFS), and time to progression (TTP) [8]. ALC/AMC-DX prognostic biomarker has subsequently been confirmed not only as a prognostic factor for survival but also correlates with tumor-associated macrophages in cHL affecting survival, suggesting an association of the biological response observed in the macroenvironment (peripheral blood-ALC/AMC) and microenvironment (tumor-associated macrophages) [9]. Thus, we studied if the Day 100 ALC/AMC ratio is a prognostic factor for cHL patients in complete remission at day 100 in a landmark analysis for OS and PFS from day 100 post-APBHSCT.

## 2. Patients and Methods

### 2.1. Patient Population

Patients were required to have undergone APBHSCT and achieved a complete remission at day 100. Patients transplanted with bone marrow or combined bone marrow and peripheral blood stem cells and patients with evidence of progression or relapse at day 100 were excluded. From 2000 to 2010, 131 consecutive cHL patients achieving a complete remission at day 100 post-APBHSCT qualified for the study. No patients refused authorization to use their medical records for research and none were lost to followup. Approval for the retrospective review of these patients' records was obtained from the Mayo Clinic Institutional Review Board, and the research was conducted in accordance with USA federal regulations and the Declaration of Helsinki.

### 2.2. End Points

The primary end-point of the study was to assess the impact of Day 100 ALC/AMC ratio on OS and PFS by landmark analysis from day 100 in cHL patients treated with APBHSCT. The Day 100 ALC, Day 100 AMC, and Day 100 ALC/AMC ratio were calculated from the Day 100 complete blood cell count (CBC) [10] obtained at day 100 followup from APBHSCT. The Day 100 ALC/AMC ratio was obtained by dividing the Day 100 ALC by the Day 100 AMC [10].

### 2.3. Prognostic Factors

The prognostic factors evaluated included the International Prognostic Score (IPS) [11] (Age, Albumin, ALC, hemoglobin, male gender, stage 4, and white blood cell (WBC) count), tumor size (≥10 cm), limited versus advanced stage, Day 15 ALC [12], Day 100 ALC, Day 100 AMC, Day 100 absolute neutrophil count, Day 100 hemoglobin, Day 100 white blood cell count, Day 100 platelets, Day 100 age, gender, and Day 100 ALC/AMC ratio.

### 2.4. Response Criteria

Definitions of response criteria, OS, and PFS were based on the guidelines from the International Harmonization Project in Lymphoma [13]. OS and PFS were evaluated from day 100 post-APBHSCT.

### 2.5. Conditioning Regimen and Stem Cell Source

All patients received BEAM BCNU (300 mg/m^2^) on day 6; Etoposide (100 mg/m^2^) and ARA-C (100 mg/m^2^) twice daily from day 5 to day 2; Melphalan (140 mg/m^2^) on day 1. All patients were infused with collected peripheral blood stem cells.

### 2.6. Statistical Analysis

Overall survival (OS) and progression-free survival (PFS) were analyzed using the approach of Kaplan and Meier [14]. Differences between the survival curves were tested for statistical significance using the two-tailed log-rank test. The Cox proportional hazard model [15] was used for the univariate and multivariate analyses to evaluate the variables under the prognostic factors section to assess their impact on OS and PFS. The choice of the best cut-off values of Day 100 ALC, Day 100 AMC, and Day 100 ALC/AMC ratio for assessing survival was based on their utility as a marker for the clinically relevant binary outcome of death/survival using the receiver operating characteristic curve (ROC) and area under the curve (AUC). The binary clinical outcome (death/survival) was established at 5 years after day 100 post-APBHSCT. Patients were classified as “alive/censored” when the follow-up time was greater than 5 years and “death” for patients known to have died before this time point [16]. A *k*-fold cross-validation with *k* values of 10 was performed to validate the results of Day 100 ALC, Day 100 AMC, and Day 100 ALC/AMC ratio [17]. Randomly chosen subsets containing 90% of the cohort were used for training and the remaining 10% were left for testing. The cross-validation process was then repeated ten times. Based on this analysis, a cross-validation AUC by the ROC was produced, representing the discriminating accuracy of Day 100 ALC, Day 100 AMC, and Day 100 ALC/AMC ratio for the binary clinical outcome of death/survival.

 Chi-square tests were used to determine relationships between categorical variables. The Wilcoxon-rank test was used to determine associations between continuous variables and categories, and Spearman's correlation coefficients were used to evaluate associations for continuous variables. All *P* values are two-sided and *P* values less than 0.05 are considered statistically significant.

## 3. Results

### 3.1. Patients Characteristics

The median age at day 100 post-APBHSCT for the cohort of 131 cHL patients was 45 years (range: 18–69 years). The distribution of additional baseline Day 100 characteristics for the 131 cHL patients in complete remission at day 100 is presented in Table 1. The median followup from day 100 post-APBHSCT was 4.1 years (range: 0.2–12.3 years) and for the living patients (*n* = 99) was 5.1 years (range: 0.4–12.3 years). Thirty patients died secondary to relapsed cHL. Two patients died of causes not related to cHL. One patient died of acute respiratory distress syndrome, and the other patient died from secondary malignancy: acute myelogenous leukemia. 

### 3.2. Cutoff for Day 100 ALC and Day 100 AMC

**Figure 1:**
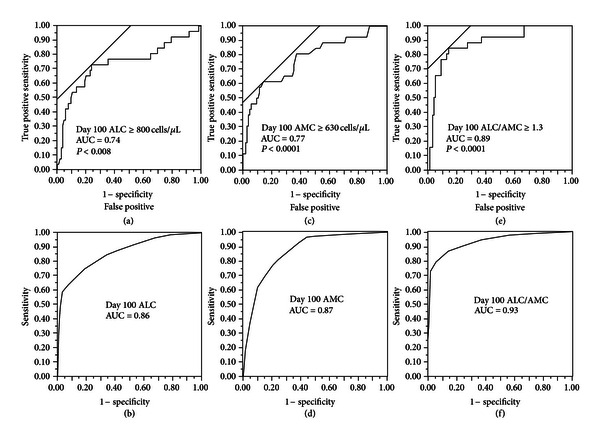
(a) Receiver operating characteristic curve (ROC) and area under the curve (AUC) for Day 100 absolute lymphocyte count (ALC). (b) *k*-fold cross-validation ROC and AUC for Day 100 ALC. (c) ROC and AUC for Day 100 absolute monocyte count (AMC). (d) *k*-fold cross validation ROC and AUC for Day 100 AMC. (e) ROC and AUC for Day 100 absolute lymphocyte count/absolute monocyte count (ALC/AMC). (f) *k*-fold cross validation ROC and AUC for Day 100 ALC/AMC.

Day 100 ALC ≥ 800 cells/*μ*L had an AUC of 0.74 (95% confidence interval (CI), 0.68 to 0.80) with a sensitivity of 73% (95% CI, 52% to 88%) and specificity of 76% (95% CI, 67% to 84%) (Figure 1(a)). Day 100 AMC ≥ 630 cells/*μ*L had an AUC of 0.77 (95% CI, 0.71 to 0.84) with a sensitivity of 62% (95% CI, 41% to 79%) and specificity of 86% (95% CI, 77% to 92%) (Figure 1(c)). Day 100 ALC/AMC ≥ 1.3 had an AUC of 0.89 (95% CI, 0.83 to 0.95) with a sensitivity of 85% (95% CI, 64% to 95%) and specificity of 87% (95% CI, 78% to 92%) (Figure 1(e)). An internal validation of Day 100 ALC, Day 100 AMC, and Day 100 ALC/AMC performances as markers for the clinical binary outcome of death/survival was performed using *k*-fold cross-validation with *k* = 10. We obtained an average AUC of 0.86 (95% CI, 0.80–0.91) over ten validation sets for Day 100 ALC with a standard deviation of 0.02; average AUC of 0.87 (95% CI, 0.82 to 0.93) over ten validation sets for Day 100 AMC with a standard deviation of 0.03; an average AUC of 0.93 (95% CI, 0.88–0.97) over ten validations sets for Day 100 ALC/AMC. We report the ROC for the complete database used in the 10-fold procedure, by collecting Day 100 (Figure 1(b)), Day 100 AMC (Figure 1(d)), and Day 100 ALC/AMC (Figure 1(f)) obtained on each fold. The closed area under the curves from the empirical ROC and the cross-validation ROC support the use of Day 100 ALC ≥ 800 cells/*μ*L, Day 100 AMC ≥ 630 cells/*μ*L, and Day 100 ALC/AMC ≥ 1.3 as the cut-off values as markers of the binary clinical outcomes of death/survival. In order to evaluate the relevance of Day 100 ALC/AMC post-APBHSCT, cHL patients were divided into two groups: Day 100 ALC/AMC ≥ 1.3 versus Day 100 ALC/AMC < 1.3 (Table 2). The only differences between the groups were gender, albumin, bulky disease, ALC at diagnosis, WBC at diagnosis, IPS, Day 100 ALC, and Day 100 AMC.

### 3.3. Day 100 ALC, Day 100 AMC, and Day 100 ALC/AMC and Survival

**Figure 2:**
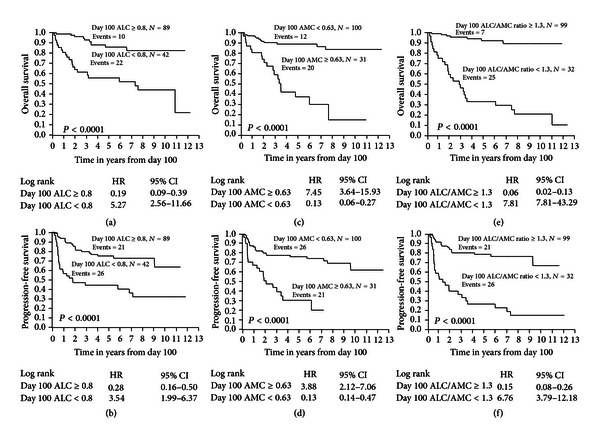
(a) Overall survival for Day 100 absolute lymphocyte count (ALC). (b) Progression-free survival for Day 100 ALC. (c) Overall survival for Day 100 absolute monocyte count (AMC). (d) Progression-free survival for Day 100 AMC. (e) Overall survival for Day 100 absolute lymphocyte count/absolute monocyte count (ALC/AMC). (f) Progression-free survival for Day 100 ALC/AMC.

Patients with a Day 100 ALC ≥ 800 cells/*μ*L experienced superior OS (Figure 2(a)) and PFS (Figure 2(b)) compared with patients with a Day 100 ALC < 800 cells/*μ*L post-APBHSCT (OS: median, not reached versus 7.4 years, 5 years OS rates of 86% (95% CI, 74% to 93%) versus 56% (95% CI, 41% to 70%), *P* < 0.0001, respectively; PFS, median, not reached versus 1.7 years, 5 years PFS rates of 76% (95% CI, 66% to 87%) versus 45% (95% CI, 33% to 61%), *P* < 0.0001, resp.). Patients with a Day 100 AMC < 630 cells/*μ*L experienced also superior OS (Figure 2(c)) and PFS (Figure 2(d)) compared with patients with a Day 100 AMC ≥ 630 cells/*μ*L post-APBHSCT (OS: median, not reached versus 2.4 years, 5-years OS rates of 89% (95% CI, 81% to 94%) versus 37% (95% CI, 21% to 57%), *P* < 0.0001, resp.; PFS, median, not reached versus 2.0 years, 5 years PFS rates of 77% (95% CI, 67% to 84%) versus 31% (95% CI, 17% to 51%), *P* < 0.0001, resp.). Patients with a Day 100 ALC/AMC ≥ 1.3 also experienced superior OS (Figure 2(e)) and PFS (Figure 2(f)) compared with patients with a Day 100 ALC/AMC < 1.3 (OS: median, not reached versus 2.8 years, 5 years OS rates of 93% (95% CI, 83% to 97%) versus 35% (95% CI, 19% to 51%), *P* < 0.0001, resp.; PFS, median, not reached versus 1.2 years, 5 years PFS rates of 79% (95% CI, 69% to 86%) versus 27% (95% CI, 14% to 45%), *P* < 0.0001, resp.). Seven patients died in the Day 100 ALC/AMC ≥ 1.3 group: 5 due to relapse cHL and 2 due to other causes. In the Day 100 ALC/AMC < 1.3 group, all 25 patients died due to relapse of cHL.

### 3.4. Univariate and Multivariate Analysis

Gender, albumin, ALC at diagnosis, hemoglobin at diagnosis, WBC at diagnosis, IPS, Day 15 ALC, Day 100 ALC, Day 100 AMC, and Day 100 ALC/AMC were predictors for OS; albumin, ALC at diagnosis, hemoglobin at diagnosis, WBC at diagnosis, IPS, Day 15 ALC, Day 100 ALC, Day 100 AMC, and Day 100 ALC/AMC were predictors for PFS by univariate analysis (Table 3). In the multivariate analysis, Day 100 ALC/AMC remained an independent predictor for OS and PFS (Table 4). Day 15 ALC was borderline statistically significant for OS and independent predictor for PFS.

## 4. Discussion

To our knowledge, there are no studies available to advise cHL patients in complete remission at day 100 of their long-term prognosis starting at day 100 post-APBHSCT. Therefore, we studied Day 100 ALC/AMC as prognostic factor for OS and PFS in a landmark analysis in cHL patients in complete remission starting at day 100 post-APBHSCT.

 To support the hypothesis that the biomarker Day 100 ALC/AMC affects survival in cHL patients, it was necessary to demonstrate that both Day 100 ALC and Day AMC were associated with clinical outcomes in cHL patients in complete remission at day 100 post-APBHSCT. We determined that cHL patients presenting with a Day 100 ALC ≥ 800 cells/*μ*L had superior survival. Similarly, cHL patients with a Day 100 AMC < 630 cells/*μ*L presented with superior survival from day 100 post-APBHSCT. This is the first paper reporting the association between Day 100 ALC and Day 100 AMC and survival in cHL patients in complete remission at day 100 post-APBHSCT. Because both Day 100 ALC and Day 100 AMC were predictors for OS and PFS, we combined them into a single prognostic factor: Day 100 ALC/AMC. A Day 100 ALC/AMC ≥ 1.3 was associated with superior OS and PFS. In the multivariate analysis, Day 100 ALC/AMC remained an independent predictor for OS and PFS. Furthermore, patients with a Day 100 ALC/AMC < 1.3 tended to have adverse features at diagnosis including high tumor burden (bulky disease), low albumin, high WBC, lymphopenia, male gender, higher IPS index, and low Day 15 ALC, suggesting the impact of host immunity (i.e., ALC) versus tumor micro-environment (i.e., AMC) on tumor growth control.

 To minimize the inherent biases of a retrospective study, the following steps were taken. With regards to selection bias we only included cHL patients that underwent APBHSCT. Patients infused with peripheral as well as bone marrow harvested stem cell were excluded. All patients were treated with the same conditioning regimen. All patients were required to be in complete remission at day 100 for the landmark analysis. With regards to confounding factors, our study included currently known prognostic factors such as IPS, tumor size; in addition, we included Day 100 ALC, Day 100 AMC, and Day 100 platelets that have been reported in the allogeneic stem cell transplant.

 The strengths of the study included a long-term followup of a well-defined group of cHL patients with complete remission at day 100 post-APBHSCT. The median followup for the cohort group was 4.1 years and 5.1 years for living patients. Secondly, Day 100 ALC/AMC combines the clinical surrogate biomarkers for the inflammatory, pathological biomarkers-tumor-infiltrating lymphocytes [18], and tumor-associated macrophages [19], which directly affect the biology of cHL. Thirdly, Day 100 ALC/AMC is a simple biomarker obtained from a CBC that can be used to asses clinical outcomes in cHL patients in complete remission at day 100 post-APBHSCT, whereas other prognostic techniques such as gene-expression profiling required fresh frozen tissue samples, limiting its use in complete remission patients.

## 5. Conclusion

 In conclusion, Day 100 ALC/AMC is a simple, low-cost predictive biomarker for prognosis in complete remission cHL patients at day 100 post-APBHSCT.

## Figures and Tables

**Table 1:** Baseline patient characteristics.

Characteristics	*N* (%)	Median	Range
*At diagnosis *			
Age, years	131 (100%)	32	(18–68)
Gender			
Male	67 (51%)		
Female	64 (49%)		
Albumin (g/dL)	127 (97%)	4	(2.0–4.6)
ALC × 10^9^/L	131 (100%)	1.3	(0.2–2.9)
Hemoglobin (g/dL)	131 (100%)	12.2	(7.7–16.2)
Mediastinal disease			
≥10 cm	21 (16%)		
<10 cm	110 (84%)		
Stage			
I	6 (4%)		
II	56 (43%)		
III	35 (27%)		
IV	34 (26%)		
Stage			
Limited	61 (47%)		
Advanced	69 (53%)		
WBC × 10^9^/L	131 (100%)	8.3	(1.4–22.2)
IPS risk factors			
Age, years			
≤45	109 (83%)		
>45	22 (17%)		
Albumin (g/dL) (*N* = 127)			
≥4	80 (63%)		
<4	47 (37%)		
ALC × 10^9^/L			
≥0.6	106 (81%)		
<0.6	25 (19%)		
Hemoglobin (g/dL)			
>10.5	102 (78%)		
≤10.5	29 (22%)		
Male	67 (51%)		
Stage 4	34 (26%)		
WBC × 10^9^/L			
>15	20 (15%)		
≤15	111 (85%)		
IPS score			
0	30 (23%)		
1	41 (31%)		
2	27 (21%)		
3	13 (10%)		
4	10 (8%)		
5	7 (5%)		
6	3 (2%)		
IPS index			
≥3	33 (25%)		
<3	98 (75%)		
*At transplant *			
Infused CD34+ stem cell × 10^6^/kg	131 (100%)	5.1	(2.1–15.8)
Pretransplant response			
CR	30 (23%)		
PR	101 (77%)		
*At Day 15 post-APBHSCT *			
ALC × 10^9^/L	131 (100%)	0.67	(0.1–3.67)
ALC × 10^9^/L			
≥0.5	90 (69%)		
<0.5	41 (31%)		
*At day 100 post-APBHSCT *			
Age, years	131 (100%)	35	(18–69)
Hemoglobin (g/dL)	131 (100%)	11.9	(7.3–16.7)
WBC × 10^9^/L	131 (100%)	4.4	(1.5–14.3)
ANC × 10^9^/L	131 (100%)	2.54	(1.2–10.01)
ALC × 10^9^/L	131 (100%)	1.09	(0.15–4.61)
AMC × 10^9^/L	131 (100%)	0.46	(0.15–4.02)
ALC/AMC	131 (100%)	2.5	(0.18–11.07)
Plts × 10^9^/L	131 (100%)	164	(55.2–373)

Abbreviations: ALC: absolute lymphocyte count; ANC: absolute neutrophil count; AMC: absolute monocyte count; CR: complete remission; IPS: International Prognostic Score; PR: partial response; Plts: platelets; and WBC: white blood cell count.

**Table 2:** Baseline characteristics based on Day 100 ALC/AMC.

Characteristics	Day 100 ALC/AMC ≥ 1.3 (*N* = 99)	Day 100 ALC/AMC < 1.3 (*N* = 32)	*P* value
*At diagnosis *			
Age, years, median (range)	33 (18–68)	32 (18–56)	0.8
Gender			<0.008
Male	44 (44%)	23 (72%)	
Female	55 (56%)	9 (28%)	
Albumin (g/dL)	4 (2–4.6)	3.9 (2.2–4.6)	<0.008
ALC × 10^9^/L	1.7 (0.2–2.9)	0.5 (0.2–2.7)	<0.0001
Hemoglobin (g/dL)	12.6 (7.7–16.2)	11.1 (8.1–15.4)	0.07
Mediastinal disease			<0.004
≥10 cm	10 (10%)	4 (34%)	
<10 cm	89 (90%)	21 (66%)	
Stage			0.9
I	5 (5%)	1 (3%)	
II	42 (43%)	14 (44%)	
III	26 (26%)	9 (28%)	
IV	26 (26%)	8 (25%)	
Stage			0.9
Limited	46 (47%)	15 (47%)	
Advanced	52 (53%)	17 (53%)	
WBC × 10^9^/L	8.0 (1.4–22.2)	12.1 (2.3–20.1)	<0.0003
IPS risk factors			
Age, years			0.9
≤45	82 (83%)	27 (84%)	
>45	17 (17%)	5 (16%)	
Albumin (g/dL) (*N* = 127)			<0.002
≥4	68 (71%)	12 (39%)	
<4	28 (29%)	19 (61%)	
ALC × 10^9^/L			<0.0001
≥0.6	92 (93%)	14 (44%)	
<0.6	7 (7%)	18 (56%)	
Hemoglobin (g/dL)			0.06
>10.5	81 (82%)	21 (66%)	
≤10.5	18 (18%)	11 (34%)	
Male	44 (44%)	23 (72%)	<0.008
Stage 4	26 (26%)	8 (25%)	0.9
WBC × 10^9^/L			<0.001
>15	9 (9%)	11 (34%)	
≤15	90 (91%)	21 (66%)	
IPS score			<0.01
0	26 (26%)	4 (13%)	
1	33 (34%)	8 (25%)	
2	21 (21%)	6 (18%)	
3	10 (10%)	3 (9%)	
4	6 (6%)	4 (13%)	
5	3 (3%)	4 (13%)	
6	0 (0%)	3 (9%)	
IPS index			<0.009
≥3	19 (19%)	14 (44%)	
<3	80 (81%)	18 (56%)	
*At transplant *			
Infused CD34+ stem cells × 10^6^/kg	5.3 (2.6–15.8)	4.5 (2.1–13.2)	0.1
Pretransplant response			0.06
CR	27 (27%)	3 (9%)	
PR	72 (72%)	29 (91%)	
ALC × 10^9^/L	0.78 (0.1–3.67)	0.39 (0.18–3.19)	<0.0001
ALC × 10^9^/L			<0.0001
≥0.5	80 (81%)	10 (31%)	
<0.5	19 (19%)	22 (69%)	
*At day 100 post-APBHSCT *			
Age, years	36 (18–66)	34.5 (19–58)	0.9
Hemoglobin (g/dL)	12.1 (8.5–16.7)	11.4 (7.3–14.1)	0.4
WBC × 10^9^/L	4.6 (2.2–9.4)	3.7 (1.5–14.3)	0.1
ANC × 10^9^/L	2.6 (0.8–6.9)	2.4 (1.0–10.0)	0.9
ALC × 10^9^/L	1.33 (0.51–4.61)	0.39 (0.15–3.94)	<0.0001
AMC × 10^9^/L	0.43 (0.15–1.58)	0.63 (0.29–4.02)	<0.0001
Plts × 10^9^/L	172 (121–373)	163 (177–334)	0.2

Abbreviations: ALC: absolute lymphocyte count; ANC: absolute neutrophil count; AMC: absolute monocyte count; CR: complete remission; IPS: international prognostic score; PR: partial response; Plts: platelets; and WBC: white blood cell count.

**Table 3:** Univariate analysis overall survival and progression-free survival.

Variables	Overall survival			Progression-free survival		
	HR	95% CI	*P*	HR	95% CI	*P*
*At diagnosis *						
Age > 45 years	1.01	0.34–2.42	0.9	0.87	0.44–1.98	0.7
Female versus male	0.34	0.15–0.72	<0.004	0.62	0.34–1.10	0.1
Albumin ≥ 4	0.30	0.14–0.61	<0.0009	0.38	0.21–0.69	<0.001
ALC ≥ 0.6 cells/*µ*L	0.12	0.06–0.24	<0.0001	0.25	0.14–0.45	<0.0001
Mediastinal ≥ 10 cm	2.06	0.93–4.26	0.07	1.87	0.93–3.51	0.08
Hemoglobin < 10.5 g/dL	3.31	1.61–6.72	<0.002	2.16	1.15–3.89	<0.02
Stage 4	1.14	0.52–2.86	0.3	1.37	0.71–2.51	0.3
WBC > 15 cells/*µ*L	4.68	2.24–9.49	<0.0001	3.18	1.64–5.84	<0.001
IPS ≥ 3	4.20	2.07–8.66	<0.0001	2.89	1.59–5.14	<0.0006
Limited versus advanced stage	0.92	0.45–1.86	0.8	0.73	0.40–1.30	0.3
Infused CD34+ stem cells	0.83	0.65–1.04	0.1	0.96	0.80–1.12	0.6
Pretransplant response (CR versus PR)	0.54	0.16–1.39	0.2	0.49	0.19–1.07	0.08
*At day 15 post-APBHSCT *						
ALC ≥ 0.5 cells/*µ*L	0.12	0.05–0.25	<0.0001	0.21	0.11–0.37	<0.0001
*At day 100 post-APBHSCT *						
Age, continuous	0.99	0.97–1.03	0.9	0.95	0.87–1.11	0.5
ALC, continuous	0.33	0.16–0.61	<0.0001	0.48	0.29–0.75	<0.0006
ALC ≥ 0.8 cells/*µ*L	0.28	0.15–0.59	<0.0001	0.28	0.16–0.50	<0.0001
AMC, continuous	2.16	1.46–2.92	<0.0001	1.80	1.24–2.40	<0.0001
AMC ≥ 0.63 cells/*µ*L	7.55	3.64–15.93	<0.0001	3.88	2.12–7.06	<0.0001
ALC/AMC, continuous	0.03	0.01–0.10	<0.0001	0.49	0.37–0.63	<0.0001
ALC/AMC ≥ 1.3	0.07	0.03–0.15	<0.0001	0.18	0.10–0.32	<0.0001
ANC, continuous	1.10	0.86–1.37	0.5	1.01	0.81–1.22	0.9
Hgb, continuous	0.93	0.76–1.13	0.5	0.96	0.82–1.13	0.7
WBC, continuous	1.06	0.89–1.22	0.5	1.00	0.86–1.14	0.9
Plts, continuous	0.91	0.87–1.11	0.2	0.97	0.90–1.17	0.3

Abbreviations: ALC: absolute lymphocyte count; ANC: absolute neutrophil count; AMC: absolute monocyte count; CR: complete remission; IPS: international prognostic score; PR: partial response; Plts: platelets; WBC: white blood cell count.

**Table 4:** Multivariate analysis overall survival and progression-free survival.

Variables at day 100 post-APBHSCT	Overall survival			Progression-free survival		
	HR	95% CI	*P*	HR	95% CI	*P*
Female versus male	0.74	0.28–1.87	0.9			
Albumin ≥ 4	0.98	0.31–3.71	0.9	0.82	0.32–1.95	0.7
ALC ≥ 0.6 cells/*µ*L	0.42	0.14–1.30	0.1	0.57	0.24–1.41	0.2
Hemoglobin < 10.5 g/dL	1.24	0.33–4.54	0.7	1.06	0.41–2.69	0.9
IPS ≥ 3	1.83	0.45–8.17	0.4	2.02	0.67–6.40	0.2
WBC > 15 cells/*µ*L	1.28	0.32–4.78	0.7	1.30	0.47–3.65	0.6
Day 15 ALC ≥ 0.5 cells/*µ*L	0.29	0.07–1.12	0.07	0.46	0.21–0.99	<0.05
Day 100 ALC ≥ 0.8 cells/*µ*L	0.55	0.20–1.42	0.2	0.54	0.25–1.16	0.1
Day 100 AMC ≥ 0.63 cells/*µ*L	1.83	0.71–4.91	0.2	1.65	0.65–4.23	0.3
Day 100 ALC/AMC ≥ 1.3	0.33	0.12–0.85	<0.02	0.37	0.18–0.72	<0.004

Abbreviations: ALC: absolute lymphocyte count; AMC: absolute monocyte count; IPS: international prognostic score; WBC: white blood cell count.
